# The psychological foundations of moral disengagement: the dynamic relationships between state desperation, psychological flexibility, and mental well-being

**DOI:** 10.3389/fpsyg.2025.1716411

**Published:** 2026-01-05

**Authors:** Mehmet Behzat Turan, Mustafa Can Koc, Osman Pepe, Laurentiu-Gabriel Talaghir, Iulia Petronela Barna, Cristina Corina Bentea, Vesile Sahiner Guler, Gabriel Marian Manolache

**Affiliations:** 1 Department of Recreation, Faculty of Sports Sciences, Erciyes University, Kayseri, Türkiye; 2 Department of Recreation, Faculty of Sports Sciences, Istanbul Gelişim University, İstanbul, Türkiye; 3 Department of Sports Management, Faculty of Sports Sciences, Süleyman Demirel University, Isparta, Türkiye; 4 Faculty of Physical Education and Sport, Dunarea de Jos University of Galati, Galati, Romania; 5 Department of Physical Education and Sports, Institute of Health Sciences, Erciyes University, Kayseri, Türkiye

**Keywords:** football players, state desperation, psychological flexibility, mental well-being, moral disengagement, competitive sport

## Abstract

**Background:**

Moral disengagement has been linked to unethical behaviors in sport, yet the mechanisms through which acute psychological states, such as state desperation, influence these behaviors remain underexplored, particularly among professional athletes.

**Objective:**

This study investigated the serial mediating role of psychological flexibility and mental well-being levels in the relationship between state desperation and moral disengagement behaviors among professional footballers.

**Methods:**

The study was a cross-sectional study and included 474 voluntarily participating professional football players who played in professional leagues across Turkey in the 2024–2025 season and were selected by a convenience sampling method. Participants completed a demographic form and scales assessing state desperation, psychological flexibility, mental well-being, and moral disengagement. Data were analyzed using SPSS v22. Pearson correlation analysis was used to determine the relationships between the variables, and regression analysis was used to assess the effect of state desperation on moral disengagement. Regression analysis based on the indirect effect approach, using the bootstrap method, was performed with the PROCESS v3.5 macro. Model 6 assessed the serial mediating effects of psychological flexibility and mental well-being. The 95% confidence interval values obtained from this analysis should not include zero.

**Results:**

The study found a negative relationship between state desperation and psychological flexibility, indicating that higher desperation is associated with reduced flexibility. Additionally, psychological flexibility was positively associated with mental well-being, while moral disengagement was positively correlated with feelings of desperation. These results suggest that individuals with a tendency to engage in desperate behavior are more likely to justify unethical actions, and improving psychological flexibility can enhance both mental health and ethical decision-making.

**Conclusion:**

The study highlights the importance of enhancing football players’ psychological flexibility and mental health to reduce moral disengagement and promote more ethical decision-making. It also shows that age, experience, and position have a significant impact on players’ performance and decision-making, underscoring the need for individual and team performance interventions.

## 1 Introduction

Sport contributes to individuals’ physical, mental, and social development while encompassing various activities focused on competition and performance. Within the spectrum of sports, football stands out as one of the most popular and heavily invested disciplines in terms of its global audience and widespread participation levels. Football’s central position makes performance metrics and success criteria even more critical. The high level of competition required in football, the game’s variable dynamics, and intense performance expectations create significant psychological pressures on players, including the pressure of winning and losing, as well as fan pressure before and after matches (Feder and Zulli, 2024).

Achieving success in the face of such pressures is also closely linked to psychological factors such as players’ ability to cope with stress, maintain motivation, and demonstrate mental resilience. Positive responses to these factors can enhance performance, while negative responses can lead to adverse outcomes and cause fluctuations in athlete performance (Karakaş and Acar, 2023). In this context, successive failures, competitions under high pressure, or a weakening sense of external control can trigger feelings of desperation in athletes.

### 1.1 State desperation

Desperation, uncertainty, and powerlessness are psychological states that arise in everyday events in human life (Garlow et al., 2008). Desperation is defined as a belief closely tied to the individual, characterized by a very weak sense of self-confidence in the social roles they find themselves in, an inability to exercise personal initiative, and the perception that they do not have control over their life (Gençöz et al., 2006). Desperation is conveyed as another negative emotion that shapes a person’s behavior in situations such as anger, shock, anxiety, and fear, which harbor negative feelings that influence a person’s behavior (Shapiro and Lie, 2004). Hopelessness is defined as an emotional state that motivates individuals to take action to resolve the problems they are currently facing and escape from distressing situations, accompanied by negative feelings (Garlow et al., 2008).

According to Hiroto and Seligman (1975), the main assumptions of the theory of desperation are as follows: As a result of the adverse events an individual experiences and their unsuccessful attempts to control these events, they begin to generalize this and see themselves as passive, exhibiting traumatic symptoms in the form of an inability to act. Alongside such symptoms, the concept of desperation is closely linked to clinical depressive characteristics, and certain psychopathological conditions are observed in individuals experiencing intense desperation (Vatan and Dağ, 2009). Garlow et al. (2008) found a positive correlation between feelings of desperation and negative emotions that individuals may experience, such as tension, anxiety, worry, anger, panic, and a sense of being out of control.

In sport, state desperation can be described as a situation in which athletes, as a result of repeatedly failing in a particular situation, believe that they cannot succeed in that specific situation. In this situation, the athlete may stop making an effort when faced with adversity that they believe is beyond their control, because they may think that whatever they do, the outcome will not change. Hannan (2019) stated that, in addition to harboring negative emotions, desperation can motivate people to commit wrongdoing. This situation may reduce athletes’ psychological flexibility.

### 1.2 Psychological flexibility

Psychological flexibility is “the ability to contact the present moment more fully as a conscious human being, and to change or persist in behavior when doing so serves valued ends” (Ciarrochi et al., 2010). Cognitive fusion and experiential avoidance are two important processes in understanding and defining psychological flexibility (Muris et al., 2017). Cognitive fusion refers to becoming entangled with one’s thoughts and interpreting them as literal realities rather than transient internal experiences (Gillanders et al., 2014). Experiential avoidance emerges as a maladaptive strategy, characterized by a set of behaviors used to avoid, escape, or control unwanted internal experiences (Hayes et al., 2006). Indeed, psychological flexibility refers to a pattern of internal processes that has proven helpful in facilitating valuable actions (Bond et al., 2011).

In sports environments, psychological flexibility can be defined as the ability to regulate internal psychological states, such as emotions, thoughts, and feelings, that might otherwise hinder an athlete’s capacity to perform effectively and meaningfully while also maintaining sportsmanship with determination. In this context, when psychological flexibility is considered within sporting environments, it involves not only managing internal states to support successful performance but also encompasses mental well-being, which is a key aspect of being mentally healthy (Tekkurşun Demir et al., 2018).

### 1.3 Mental well-being

The World Health Organization (2004) defined mental well-being as “being aware of one’s abilities, overcoming the stress in one’s life, being productive and useful in business life, and contributing to society in line with one’s abilities.” Additionally, it has been noted that mental well-being has a positive influence on individuals’ emotional states, significantly enhancing the quality of life and overall health. Research in Western societies has found that health, nutrition, and sports are closely associated with mental well-being (Diener and Chan, 2011).

It has been stated that stress, considered one of the determinants of success in sports, plays an influential role in athletes’ psychological resilience, coping strategies, and their ability to manage, reduce, recover from, and quickly return to everyday life (Nicholls et al., 2009; Codonhato et al., 2018; Cappella and Weinstein, 2001). At the same time, mental well-being is likely to help athletes cope with the various challenges they face throughout their sports careers. Although most interventions implemented and evaluated within the scope of sport psychology focus on the obstacles athletes face, they aim to directly or indirectly increase their mental well-being components (Williams, 2009). In this context, the feelings of struggle and anxiety that athletes experience in response to psychological and physiological pressures are thought to be closely related to their mental well-being.

### 1.4 Moral disengagement

The concept of moral disengagement was introduced within Albert Bandura’s Social Cognitive Theory, which posits that human behavior is influenced not only by internal cognitive and emotional processes but also by environmental and social factors (Bandura, 2002). According to Bandura et al. (1996), moral disengagement refers to a cognitive mechanism that allows individuals to justify harmful behaviors, thus reducing feelings of guilt and enabling them to engage in unethical actions repeatedly without experiencing sufficient emotional distress. This process is considered a key psychosocial factor influencing moral cognition and behavior, facilitating actions contrary to personal moral principles without the usual emotional barriers (Knoth and Javidan, 2024). At the same time, Bandura (1991) argued that a comprehensive moral theory should explain how moral reasoning and other psychosocial factors shape moral behavior. In the theory he developed in this direction, he emphasized the determining role of moral disengagement on moral behavior.

Moral disengagement has been widely studied in sport psychology as a cognitive mechanism that allows athletes to rationalize unethical or aggressive behaviors without feeling personal guilt (Bandura, 1991, 2002). Within this framework, psychological flexibility conceptualized in Acceptance and Commitment Theory (Hayes et al., 2012) has emerged as a key construct for understanding how athletes manage internal experiences, thoughts, and emotions under pressure (Birrer et al., 2012).

Athletes with greater psychological flexibility are better able to adaptively regulate their behaviors and align them with personal and ethical values, even in competitive and stressful environments. Integrating these perspectives provides a contemporary understanding of moral functioning in sport, suggesting that psychological flexibility may play a protective role against moral disengagement. This relationship, however, remains underexplored in the literature and warrants further empirical investigation, particularly among professional football players.

In competitive sports, the physical and psychological pressures of competition have been shown to trigger moral disengagement, leading athletes to engage in unethical behaviors, such as humiliation, intentional harm, and other detrimental actions (Kavussanu, 2008). These behaviors often arise under heightened stress, where athletes may feel justified in bending or abandoning their moral values to achieve success. Studies have also shown that social and environmental pressures, such as spectators, coaches, and team dynamics, can exacerbate these tendencies, fostering an environment where athletes are more likely to engage in moral disengagement (Boardley and Kavussanu, 2008; Boardley and Kavussanu, 2011). Furthermore, empirical evidence suggests that athletes facing high levels of moral disengagement may struggle with their psychological flexibility, defined as the ability to adapt to challenging situations, regulate emotional responses, and maintain psychological resilience (Hayes et al., 2012).

### 1.5 The present study

The reason for choosing football in this study is that football has evolved from being just a game into a multidimensional phenomenon that has created its industry and values. As a significant industry, the pursuit of victory both materially and spiritually is central to football.

Players frequently encounter physical and psychological difficulties in football’s challenging and high-stakes competitive environment. In such situations, their desperation can lead them toward attitudes and behaviors that deviate from ethics, both on and off the pitch. Moral disengagement refers to cognitive mechanisms that allow athletes to justify unethical behavior without experiencing internal conflict. In contrast, psychological flexibility refers to an individual’s ability to adapt effectively to stress factors and align their behavior with their values. In this context, mental well-being encompasses positive functioning and the capacity to manage competitive pressure.

This study highlights the serial mediating role of psychological flexibility and mental well-being by investigating the complex psychological interaction between state desperation and moral disengagement among football players.

In a literature review conducted by the researchers, moral disengagement was associated with antisocial behavior, aggression, and doping, particularly among young athletes (Gentile et al., 2022; Luo and Bussey, 2023). However, these studies have primarily focused on general tendencies and have not sufficiently addressed the effects of acute psychological states such as state desperation. On the other hand, psychological flexibility is negatively related to anxiety and depression (Johles et al., 2020) and positively associated with mental well-being (Ronkainen et al., 2024).

Nevertheless, no study to date has examined how state desperation influences moral disengagement through the underlying psychological mechanisms of psychological flexibility and mental well-being. Addressing this gap, the present study investigates these mediating processes among professional football players in Turkey, thereby offering both theoretical and practical contributions to the field of sport psychology. It is proposed that higher levels of state desperation are positively associated with moral disengagement behaviors (H1), while negatively predicting psychological flexibility (H2) and mental well-being (H3). Moreover, psychological flexibility (H4) and mental well-being (H5) are expected to exert negative effects on moral disengagement behaviors. In addition, psychological flexibility (H6) and mental well-being (H7) are hypothesized to mediate the relationship between state desperation and moral disengagement behaviors. Finally, it is anticipated that psychological flexibility and mental well-being will jointly function as serial mediators in the association between state desperation and moral disengagement behaviors (H8).

### 1.6 Theoretical framework and the series mediation pathway

Football players are likely to feel helpless when faced with failures in environments where stress and external factors (e.g., referee decisions, team management, and fan pressure) are present, and when these failures are repeated. In this context, state desperation is a condition characterized by passivity and hopelessness, arising from an individual’s loss of control in the face of repeated negative life experiences (Seligman, 1975).

When football players develop a cognitive generalization that external conditions do not affect them, psychological and behavioral functioning impairments may occur both on and off the pitch. This situation can lead to adverse outcomes, particularly in football players’ ethical decision-making processes. One such negative outcome is moral disengagement behavior. Bandura (1991) defines moral disengagement as the process whereby individuals temporarily suspend their ethical principles to justify their unethical behavior. Research indicates that individuals under high stress are more prone to rationalize their unethical behavior through cognitive restructuring (Moore, 2008; Fida et al., 2015; Zhong et al., 2025). When considered in the context of football, players may deny responsibility for their actions, belittle the injured party, or trivialize the consequences of their behavior. Players’ internal psychological resources can shape this relationship between state desperation and moral disengagement. Psychological flexibility and mental well-being, as these psychological resources, can be considered important mediating variables influencing this relationship. Psychological flexibility is an individual’s capacity to remain in the present moment, accept their internal experiences, and act according to their values (Hayes et al., 2012). Mental well-being refers to an individual’s psychological resilience, capacity to experience positive emotions, and satisfaction with life (Ryff, 1989; Diener et al., 2010). Psychological flexibility has been found to have positive effects on an individual’s overall mental health; individuals with high levels of mental well-being have been found to have stronger adherence to ethical principles (Kashdan and Rottenberg, 2010; Keyes and Haidt, 2003). Within this theoretical framework, it is predicted that psychological flexibility, followed by mental well-being, may play a serial mediating role in explaining the relationship between state desperation and moral disengagement experienced by football players.

The sequential mediation model proposed by the study argues that the effect of the state desperation (X) variable on moral disengagement (Y) can be indirectly explained through psychological flexibility (M1) and mental well-being (M2), respectively.

## 2 Materials and methods

### 2.1 Determination of sample size

In order to determine the appropriate sample size for the present study, a power analysis was conducted to examine the statistical power required to detect the hypothesized serial mediation effects among moral disengagement, state desperation, psychological flexibility, and mental well-being among professional football players. Following the recommendations of Hayes (2018) and Fritz and MacKinnon (2007), we based our analysis on expected standardized path coefficients representing small, medium, and significant effects. A preliminary analytical estimation was performed using the delta method for indirect effects (Sobel, 1982), which allows approximate computation of the standard error for a serial mediation path (X → M₁ → M₂ → Y). The estimated power levels were calculated under four effect-size scenarios: Small effects (a = d = b = 0.14), Medium effects (a = d = b = 0.39), Large effects (a = d = b = 0.59), and Mixed effects (a = d = 0.39, b = 0.14).

**Figure 1:**
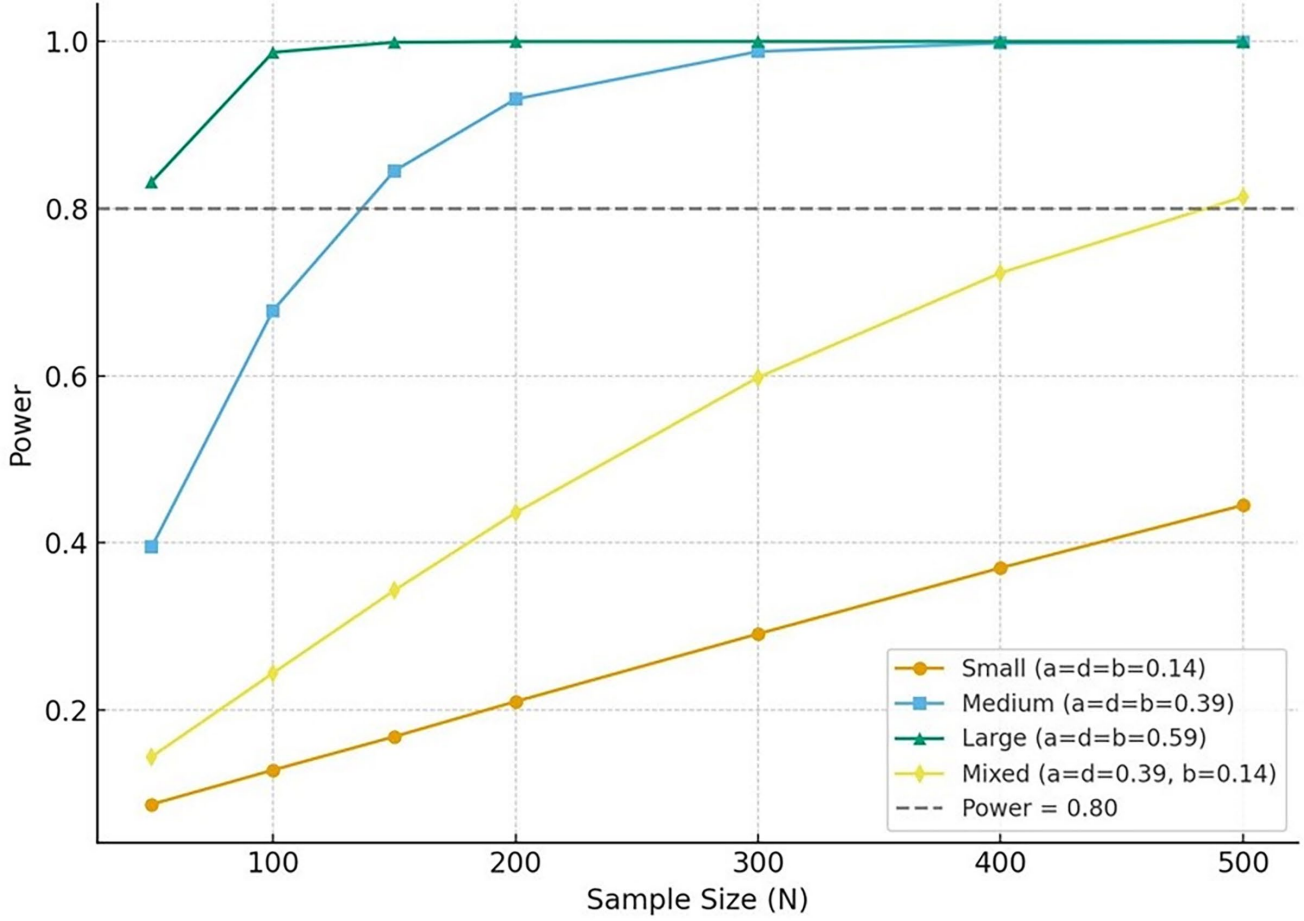
The power curves for serial mediation effects.

The results indicated that when all three path coefficients were assumed to be medium in size, a minimum sample size of approximately *N* = 140 was required to achieve a statistical power of 0.80 at *α* = 0.05. Conversely, for large effects, a sample size of *N* ≈ 50 would be sufficient. In contrast, in the mixed scenario (i.e., when the final path is small), a considerably larger sample size of *N* ≈ 490 was necessary to reach comparable power levels. Under conditions where all paths are small, achieving adequate power would require an impractically large sample, suggesting the need to assume at least moderate effect sizes for a feasible research design. The current study assumes medium effect sizes for the primary model paths based on previous research on mediation effects in psychological and sports contexts (Fritz and MacKinnon, 2007; Preacher and Hayes, 2008). Accordingly, the target sample size for this study was set at a minimum of *N* = 150 professional football players, which provides an estimated power of approximately 0.84. This sample size is deemed sufficient to reliably test the hypothesized serial mediation model and aligns with recommendations in mediation literature (Figure 1).

### 2.2 Participants

The study population consists of football players competing in 164 teams in professional leagues across the Turkish Football Federation in the 2024–2025 season. A total of 2,837 football players compete in professional football leagues in Turkey.

In the study, the convenience sampling method was used, which involves selecting readily available and willing participants, thus facilitating data collection in a practical and time-efficient manner (Etikan et al., 2016). The inclusion criteria were as follows: (i) being a Turkish citizen; (ii) being older than 18 years of age; (iii) actively playing football for at least 3 years in professional football clubs; (iv) being younger than 35 years of age; and (v) completing the voluntary informed consent form online. Football players competing in amateur leagues were excluded from the study to ensure consistency and comparability by focusing solely on professional football players. The study was limited to the characteristics specified, and data were collected only from football players who met the inclusion criteria and were accessible to the researchers.

The scales were distributed online to professional football clubs, and athletes who met the inclusion criteria and voluntarily agreed to participate completed the questionnaires.

The study collected data from as many football players as possible, and 497 participants were reached. During the data analysis, 12 participants’ questionnaires were found to be incomplete or incorrectly completed, and 11 participants were excluded from the study according to the outlier analysis. As a result, 474 football players voluntarily participated in the study, and data were collected between January 15 and February 10, 2025.

### 2.3 Research model

The convenience sampling method was used in the observational study that analyzed data from a population at a single point in time.

This correlational survey study determined the relationships between state desperation, psychological flexibility, mental well-being, and moral disengagement. Additionally, the study aimed to examine the serial mediation effects of psychological flexibility and mental well-being in the relationship between state desperation and moral disengagement among football players.

In serial multiple mediation, the effect of an independent variable on a dependent variable is transmitted through more than one mediator in a specific causal sequence. This model allows researchers to test whether mediators function as part of a causal chain linking the predictor to the outcome (Hayes, 2018; Hayes, 2022).

**Figure 2:**
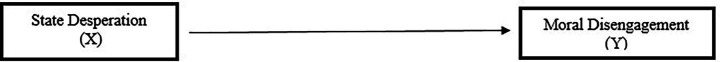
The effect of moral disengagement on mental well-being.

**Figure 3:**
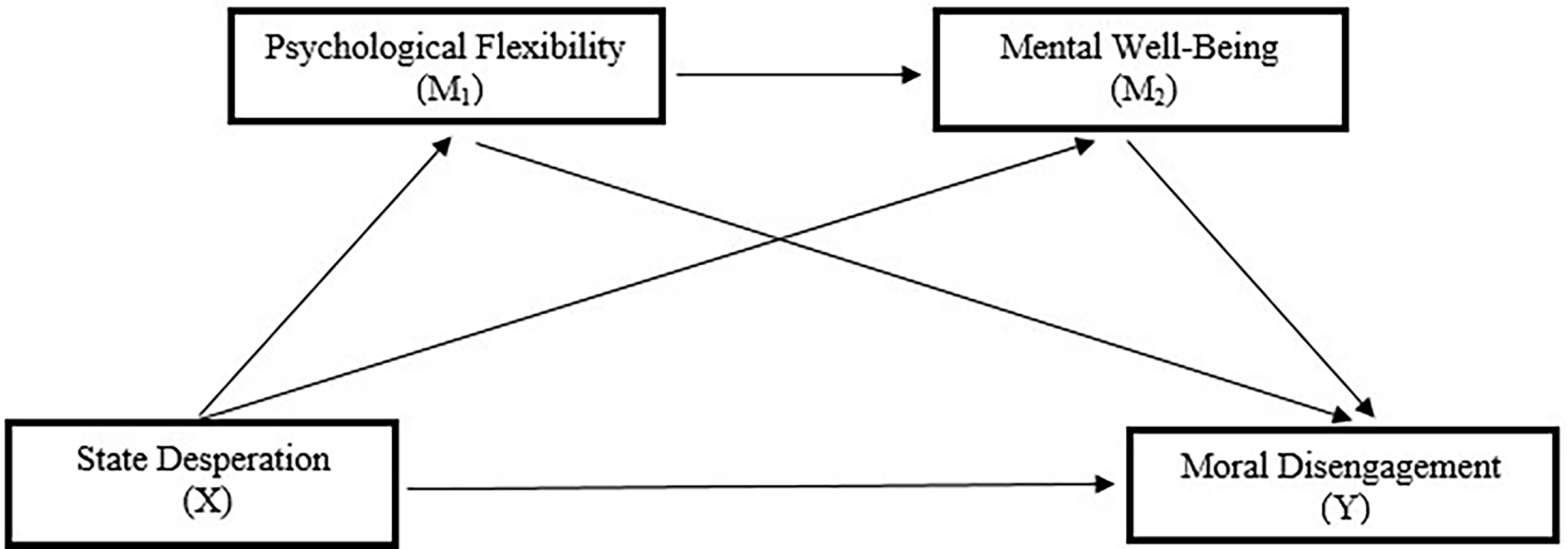
The serial mediation effects of psychological flexibility and mental well-being.

Accordingly, the serial mediation effects of psychological flexibility (M1) and mental well-being (M2) in the relationship between state desperation (X) and moral disengagement (Y) were examined in the data analysis. Figure 2 shows the effect of moral disengagement on mental well-being in football players, and Figure 3 shows the serial mediation effects of psychological flexibility and state desperation.

### 2.4 Data collection tools

Data collection for this study was conducted online using the Google Forms application. The researchers collaborated with team managers and coaches to distribute the survey link. After getting permission from team managers and coaches, a survey link was shared with football players. Participants were asked to complete a demographic information form developed by the researchers, scales taken from the literature measuring state desperation, psychological flexibility, mental well-being, and moral disengagement in sport, and a consent form confirming their voluntary participation in the study.

#### 2.4.1 Demographic information form

The researchers prepared the personal information form, which consisted of four questions designed to gather information on the participants’ age, years of experience in football, position played, and whether they had played at the national team level.

When Table 1 is examined, it can be seen that 53.2% of the football players are aged 18–23, 27.6% are 24–29, and 19.2% are 30–35. In terms of years of experience in football, 25.5% have 3–8 years of experience, 38% have 9–14 years, and 36.5% have 15 or more years of experience. Regarding playing positions, 16.7% are goalkeepers, 26.2% are defenders, 35.7% are midfielders, and 21.5% are attackers. Additionally, 11% reported it to have played at the national team level, while 89% did not.

**Table 1:** Descriptive information of participants.

Variables	Groups	*n*	%
Age	18–23	252	53.2
	24–29	131	27.6
	30–35	91	19.2
Years of experience in football	3–8	121	25.5
	9–14	180	38.0
	15+	173	36.5
Position you play	Goalkeeper	79	16.7
	Defender	124	26.2
	Midfielder	169	35.7
	Attacker	102	21.5
Played at a national level before	Yes	52	11.0
	No	422	89.0

#### 2.4.2 The state desperation scale (SDS)

The state desperation scale was developed to measure an individual’s desperation level in line with the feelings he/she has determined. The scale was developed by Hannan and Hackathorn (2022) and adapted into Turkish by Ekşi and Benli (2023). The scale adapted to Turkish culture consists of 9 items, a 9-point Likert-type scale, and two sub-dimensions. The sub-dimensions in the scale are Emotion (5 items) and Motivation (4 items). Items of Motivation sub-dimensions are reverse-scored. The minimum score is 0, and the maximum score is 81. The Cronbach’s alpha internal consistency reliability coefficient was reported as 0.80. In this study, the scale was evaluated based on the total score.

#### 2.4.3 The psychological flexibility scale (PFS)

The Psychological Flexibility Scale was developed to measure the level of behavior of an individual in line with the values he/she has determined in the moment, outside of the past and future. The scale was developed by Francis et al. (2016) and adapted into Turkish by Karakuş and Akbay (2020). The scale adapted to Turkish culture consists of 28 items, a 7-point Likert-type scale, and five sub-dimensions. The sub-dimensions in the scale are Value and Behavior in Line with Value (10 items), Being in the Moment (7 items), Acceptance (5 items), Contextual Self (3 items), and Dissociation (3 items). The high scores obtained from the sub-dimensions in the scale indicate that individuals have high psychological flexibility. Items 2, 3, 5, 6, 8, 18, 20, 22, 23, 24, and 25 are reverse-scored. The minimum score is 28, and the maximum score is 196. The Cronbach’s alpha internal consistency reliability coefficient was reported as 0.79. In this study, the scale was evaluated based on the total score.

#### 2.4.4 The Warwick-Edinburgh mental well-being scale (WEMWBS)

The Warwick-Edinburgh Mental Well-Being Scale was developed to measure individuals’ positive mental health by including psychological and subjective well-being. The scale was developed by Tennant et al. (2007) and adapted into Turkish by Keldal (2015). The scale comprises 14 positively worded items, each answered using a 5-point Likert-type scale. Scores range from a minimum of 14 to 70, with higher scores indicating greater mental well-being. The scale demonstrated excellent reliability, with a Cronbach’s Alpha internal consistency coefficient of 0.92.

#### 2.4.5 The moral disengagement in sport scale-short (MDSS-S)

The Moral Disengagement in Sport Scale was developed to measure the moral disengagement mechanisms of athletes. The short form of the moral disengagement scale in sport was developed by Boardley and Kavussanu (2008) and adapted into Turkish by Gürpınar (2015). The scale consists of eight items and employs a 7-point Likert format. All items on the scale are negatively phrased, measuring attitudes of distancing from morality in sports. Higher scores indicate that athletes are more likely to deviate from moral principles. For the scale’s reliability, Cronbach’s Alpha was calculated as 0.77.

The Cronbach alpha values obtained according to the responses of the football players participating in this study are presented in Table 2.

**Table 2:** Descriptive values of the sub-dimensions of the scales.

Scales	Item number	Cronbach’s Alpha
State desperation	9	0.780
Psychological flexibility	28	0.925
The Warwick-Edinburgh mental well-being	14	0.917
Moral disengagement in sport	8	0.922

When Table 2 is examined, Cronbach’s Alpha values indicate that the internal consistency coefficient for the State Desperation Scale is 0.780, for the Psychological Flexibility Scale is 0.925, for the Warwick-Edinburgh Mental Well-Being Scale is 0.917, and for the Moral Disengagement in Sport Scale is 0.922.

Cronbach’s Alpha is a reliability coefficient used to assess the internal consistency of multi-item scales. In other words, it is used to determine to what extent the items in a scale are related to each other, that is, whether they measure the same concept. The value range varies between 0 and 1; a value of 0.70 and above is generally interpreted as an acceptable level of internal consistency (Nunnally and Bernstein, 1994). These values demonstrate that the data provided by the participants on these scales exhibit an acceptable level of internal consistency.

The fit indices obtained from Confirmatory Factor Analysis (CFA) were used to assess the model’s suitability for the data. Considering the limits accepted in the literature, all indices were acceptable or indicated a good fit. As shown in Table 3, all scales have an acceptable level of model fit.

**Table 3:** Fit indices of scales.

Scales	χ^2^/df	CFI	TLI	RMSEA	SRMR
State desperation	2.30	0.95	0.93	0.070	0.060
Psychological flexibility	2.10	0.96	0.94	0.068	0.055
The Warwick-Edinburgh mental well-being	2.45	0.94	0.92	0.072	0.050
Moral disengagement in sport	1.80	0.97	0.96	0.060	0.040

When examining the results in Table 3, it is observed that the χ^2^/df values for all scales are below 3. This indicates that the model has an acceptable level of fit (Kline, 2016). Furthermore, CFI and TLI values above 0.90 indicate that the models have a good level of fit (Hu and Bentler, 1999). RMSEA values between 0.05 and 0.08 indicate acceptable fit, while SRMR values below 0.08 indicate good fit. Therefore, the CFI results support the construct validity of the scales used. Exploratory Factor Analysis results are provided in Appendix A.

#### 2.4.6 Analysis of data

Data was analyzed using SPSS v22. The Kolmogorov–Smirnov test, one of the tests used to assess the normality of data distributions (Berger and Zhou, 2014), was employed. The normality results of the scores obtained in this study are presented in Table 4.

**Table 4:** Skewness, Kurtosis, and Kolmogorov–Smirnov test significance level results of the participants’ scale scores.

Scales	Skewness	Kurtosis	*p*
State desperation	0.224	1.438	0.000**
Psychological flexibility	−0.005	−0.148	0.011*
The Warwick-Edinburgh mental well-being	−0.511	0.283	0.000**
Moral disengagement in sport	0.218	−0.195	0.000**

When Table 4 is examined, the skewness and kurtosis values of the data fall within the range of ±1.5. Values within ±2 (George and Mallery, 2010) are interpreted as indicating the absence of excessive deviations from normality. Consequently, the data were normally distributed and suitable for parametric tests. Pearson correlation analysis was used to determine the relationships between the variables, and the Fisher *Z* transformation test was applied to compare these relationships. Regression analysis was used to determine the effect of state desperation on moral disengagement. To assess the serial mediation effects of psychological flexibility and mental well-being, regression analysis based on the indirect effect approach using the Bootstrap method was conducted via the PROCESS v3.5 macro. The PROCESS Macro Model 6 option developed by Hayes (2013) was employed to examine the serial mediation effect, with a 5,000-resample option applied in the Bootstrap method. The 95% confidence interval values obtained from this analysis should not include zero (Gürbüz, 2019; Hayes, 2013).

## 3 Results

When Table 5 was examined, it was determined that the level of state desperation of the football players participating in the study was 21.84 ± 9.49, psychological flexibility was 130.92 ± 27.28, mental well-being was 56.06 ± 9.17, and moral disengagement was 28.14 ± 7.49. Furthermore, Fisher’s *Z* transformations for the correlations between the various scales were calculated as follows: the correlation between participants’ state desperation and their psychological flexibility (*r* = −0.379, *p* < 0.01) resulted in a *Z* score of −0.400, their mental well-being (*r* = −0.242, *p* < 0.01) resulted in a *Z* score of −0.245, and moral disengagement (*r* = 0.237, *p* < 0.01) resulted in a Z score of 0.245. The correlation between participants’ psychological flexibility and their mental well-being (*r* = 0.381, *p* < 0.01) resulted in a *Z* score of 0.400, their moral disengagement (*r* = −0.335, *p* < 0.01) resulted in a *Z* score of −0.354. The correlation between participants’ mental well-being and their moral disengagement (*r* = −0.403, *p* < 0.01) resulted in a *Z* score of −0.424.

**Table 5:** Descriptive statistics and Pearson correlation coefficients for the relationships between variables.

Scales	Min	Max	M ± SD	1	2	3	4
1. State desperation	0.00	60.00	21.84 ± 9.49	1	−0.379**	−0.242**	0.237**
2. Psychological flexibility	32.00	196.00	130.92 ± 27.28	−0.379**	1	0.381**	−0.335**
3. The Warwick-Edinburgh mental well-being	18.00	70.00	56.06 ± 9.17	−0.242**	0.381**	1	−0.403**
4. Moral disengagement in sport	10.00	48.00	28.14 ± 7.49	0.237**	−0.335**	−0.403**	1

**p < 0.01, *n* = 474, 1- State Desperation, 2- Psychological flexibility, 3- Mental well-being, 4- Moral disengagement.

When Table 6 was examined, the model shows a moderate correlation (*R* = 0.456), with 20.8% of the variance in the dependent variable explained (*R*^2^ = 0.208). The Adjusted R^2^ of 0.203 suggests a modest fit, and the Standard Error of 6.683 indicates some variability in predictions.

**Table 6:** Summary of regression model fit and prediction accuracy.

R	R^2^	Adjusted R^2^	Std. Error
0.456	0.208	0.203	6.683

When Table 7 was examined, the ANOVA results indicate that the regression model is statistically significant (*p* < 0.001). The *F*-value of 41.105 suggests that the model explains a significant portion of the variance in the dependent variable. The residual variance is 20994.016. These findings demonstrate that the model provides a meaningful contribution and shows good fit.

**Table 7:** Analysis of variance (ANOVA) for regression model.

Source	Sum of squares	df	Mean square	*F*	*p*
Regression	5508.229	3	1836.076	41.105	0.001**
Residual	20994.016	470	44.668	-	
Total	26502.245	473	-	-	

**p < 0.01.

When Table 8 was examined, the table shows significant relationships between the predictors and the dependent variable. The constant is significant (*p* < 0.001).

- State Desperation has a small positive effect (B = 0.074, *p* < 0.05).
- The Warwick-Edinburgh Mental Well-Being score has a negative effect (B = −0.254, *p* < 0.001).
- Psychological Flexibility also has a negative effect (B = −0.049, *p* < 0.001).

**Table 8:** Regression coefficients.

Variable	B	Std. Error	Beta	*p*
Constant	47.243	2.583	-	0.001**
State desperation	0.074	0.035	0.094	0.036*
The Warwick-Edinburgh mental well-being	−0.254	0.036	−0.311	0.001**
Psychological flexibility	−0.049	0.013	−0.180	0.001**

**p < 0.01, *p < 0.05.

All predictors are statistically significant, with the strongest effect from The Warwick-Edinburgh Mental Well-Being.

Table 9 presents collinearity statistics and Cook’s Distance values for three variables: State Desperation, the Warwick-Edinburgh Mental Well-Being Scale, and Psychological Flexibility. The tolerance values and Variance Inflation Factor (VIF) values are relatively low, indicating that multicollinearity is not an issue in the data. Furthermore, the Cook’s Distance values are very small, with a minimum of 0, a maximum of 0.072, and a mean close to 0 (0.003), suggesting that there are no influential outliers that could affect the regression results. Overall, the data appears to be reliable, with no significant concerns regarding multicollinearity or influential data points.

**Table 9:** Collinearity statistics and Cook’s distance.

Variable	Tolerance	VIF
State desperation	0.845	1.183
The Warwick-Edinburgh mental well-being	0.844	1.185
Psychological flexibility	0.767	1.303

	Min	Max	Mean	SD
Cook’s distance	0.000	0.072	0.003	0.007

**Figure 4:**
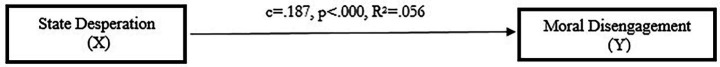
The effect of moral disengagement on mental well-being.

**Figure 5:**
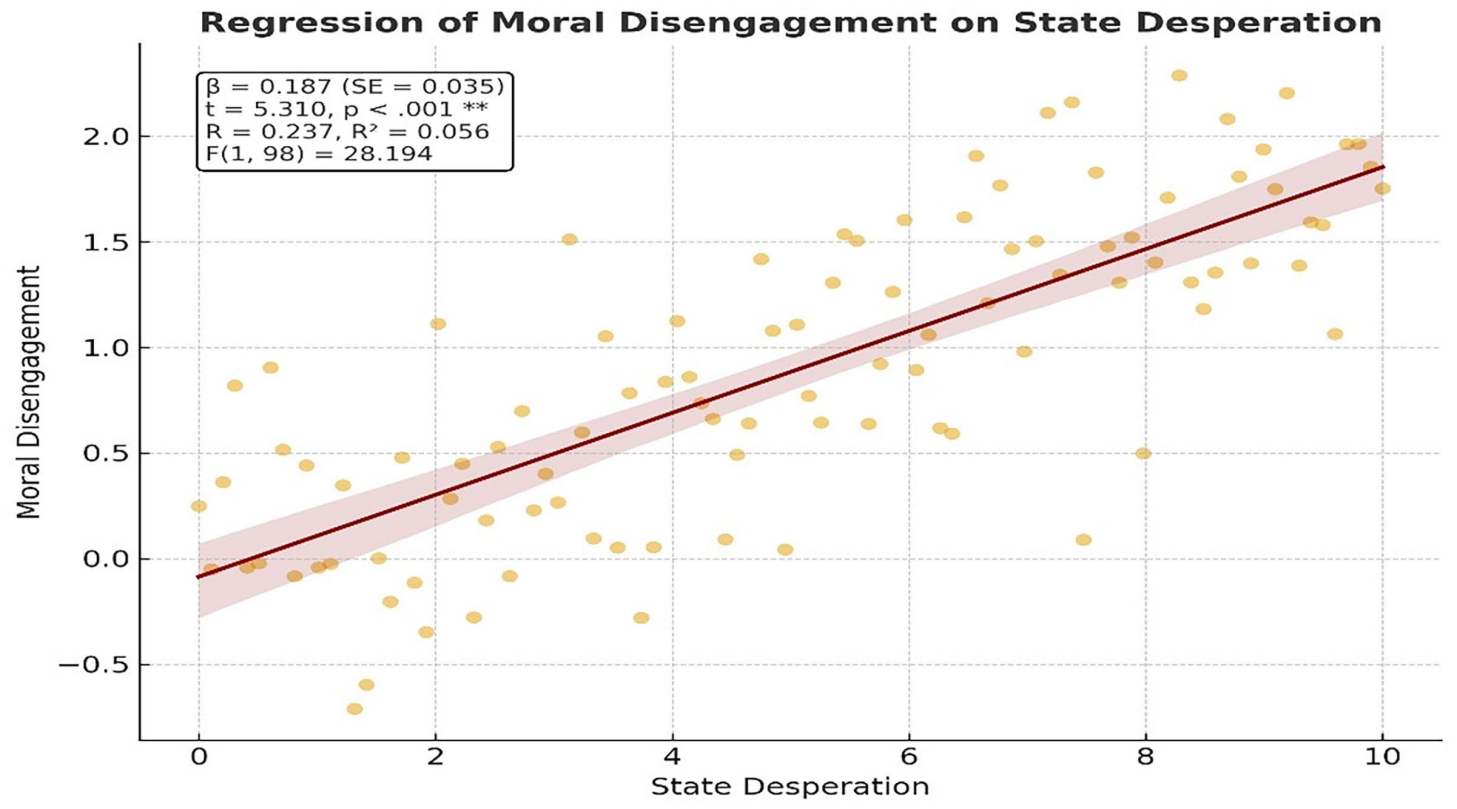
The depend variable moral disengagement histogram.

When Table 10 is examined, the regression model indicates a significant relationship between the football players’ state desperation and moral disengagement (*F* = 28.194, *p* < 0.001). According to the *t*-test results regarding the significance of the regression coefficient, it was determined that moral disengagement significantly predicts state desperation (*t* = 5.310, *p* < 0.001). This model explains 5.6% of the variance in the participants’ moral disengagement (*R*^2^ = 0.056, *p* < 0.001). Figure 4 shows the effect of moral disengagement on mental well-being in football players and Figure 5 shows the histogram of the depend variable.

**Table 10:** The effect of state desperation on moral disengagement.

Variables		β	SE	*t*	*p*	*R*	*R* ^2^	*F*	*p*
Independent	Depend								
State desperation	Moral disengagement	0.187	0.035	5.310	0.000	0.237	0.056	28.194	0.000**

**p < 0.01.

**Figure 6:**
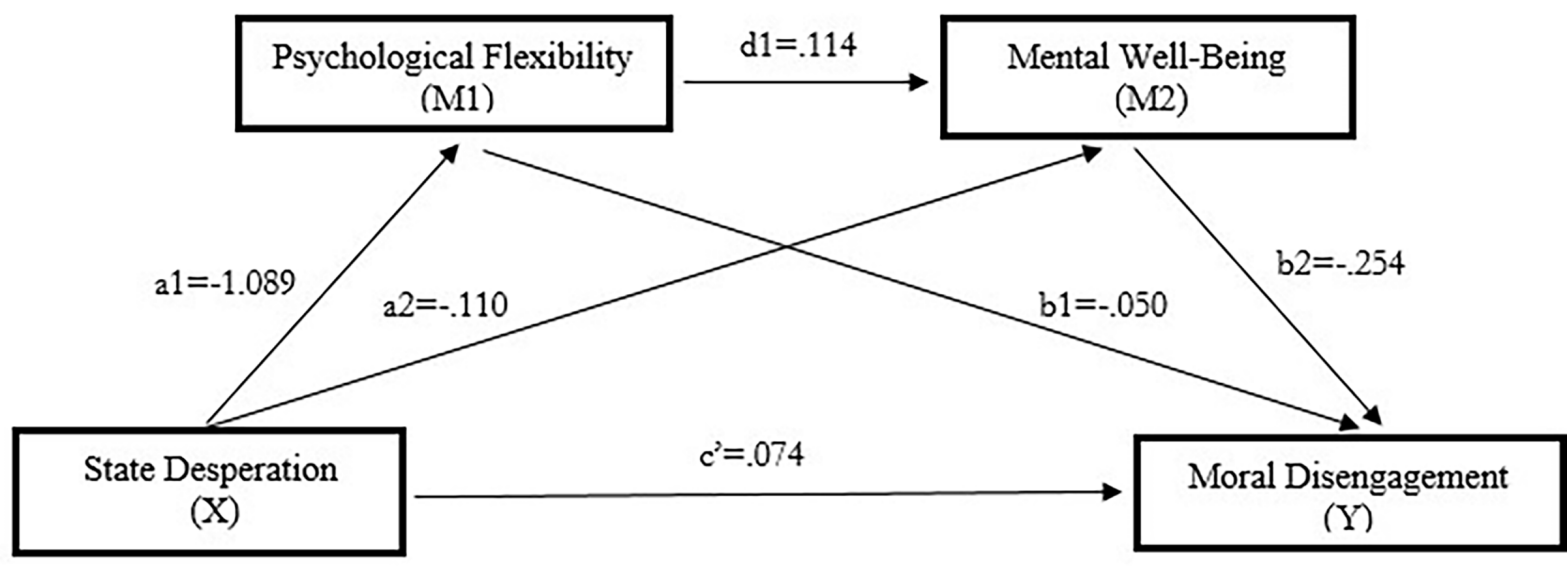
The serial mediation effects of psychological flexibility and mental well-being.

When Table 11 examined, It was determined that the state desperation variable had a negative and statistically significant effect on psychological flexibility (path a1) (a1 = −1.089, *t* = −8.898, *p* < 0.001). When the effect of psychological flexibility variable had a negative and significant effect on moral disengagement (path b1), (b1 = −0.050, *t* = −3.847, *p* < 0.001). It was also determined that the state desperation variable had a negative and statistically significant effect on mental well-being (path a2) (a2 = −0.110, *t* = −2.480, *p* < 0.001). When the effect of mental well-being variable had a negative and significant effect on moral disengagement (path b2), (b2 = −0.254, *t* = −6.960, *p* < 0.001). In addition, It was determined that the psychological flexibility variable had a positive and statistically significant effect on mental well-being (path d1) (d1 = 0.114, *t* = 0.014, *p* < 0.001). On the other hand, when the direct effect of state desperation on moral disengagement (path c’) was analyzed, it was found that this effect was positive and statistically significant (c’ = 0.074, *t* = 2.103, *p* < 0.001). Figure 6 shows the serial mediation effects of psychological flexibility and state desperation.

**Table 11:** The serial mediation role of psychological flexibility and state desperation between moral disengagement and mental well-being (*N* = 474).

Outcomes												
	Psychological flexibility (M1)				Mental well-being (M2)				Moral disengagement (Y)			
		b	SE	t		b	SE	t		b	SE	t
State desperation (X)	a1	−1.089	0.122	−8.898	a2	−0.110	0.044	−2.480	c’	0.074	0.035	2.103
Psychological flexibility (M1)	-	-	-	-	d1	0.114	0.014	7.399	b1	−0.050	0.013	−3.847
Mental well-being (M2)	-	-	-	-	-	-	-	-	b2	−0.254	0.037	−6.960
Constant		R^2^ = 0.144				R^2^ = 0.156				R^2^ = 0.208		
		*F*(1,472) = 79.167				*F*(1,472) = 43.674				*F*(1,472) = 41.105		
		*p* < 0.000**				*p* < 0.000**				*p* < 0.000**		

**p < 0.01.

As illustrated in Figure 6, serial multiple mediation model 6 was used. The model contains two mediating variables, three indirect effects and one direct effect. These effects are as follows: The indirect effect of state desperation on moral disengagement through psychological flexibility (a1b1), The indirect effect of state desperation via mental well-being on moral disengagement (a2b2), The indirect effect of state desperation on moral disengagement via psychological flexibility and mental well-being (a1d1b2), The sum of these three indirect effects represents the total indirect effect of moral disengagement (Serial mediation effect: a1b1 + a2b2 + a1d1b2).

As indicated in Table 12, it was found that state desperation has a negative and statistically significant direct effect on moral disengagement. (B = 0.113, SE = 0.023, CI = [0.071; 0.161]). The first indirect effect is the indirect effect of state desperation on moral disengagement through psychological flexibility. [Ind1 = state desperation → psychological flexibility → moral disengagement]. This indirect effect is statistically significant (B = 0.054, SH = 0.018, CI = [0.021; 0.092]). The second indirect effect is the impact of state desperation on moral disengagement through mental well-being. [Ind 2 = state desperation → mental well-being → moral disengagement]. This indirect effect is statistically significant (B = 0.028, SH = 0.012, CI = [0.005; 0.052]). The third indirect effect is the serial effect of state desperation on moral disengagement through psychological flexibility and mental well-being. [Ind 3 = state desperation → psychological flexibility → mental well-being → moral disengagement]. This indirect effect is statistically significant. (B = 0.032, SH = 0.009, CI = [0.017; 0.053]).

**Table 12:** The indirect effects of state desperation and moral disengagement (*n* = 474).

Indirect effects	b	SE	LLCI	ULCI
Total effect	0.113	0.023	0.071	0.161
Ind 1	0.054	0.018	0.021	0.092
Ind 2	0.028	0.012	0.005	0.052
Ind 3	0.032	0.009	0.017	0.053

## 4 Discussion

Football is not only physically demanding but also requires substantial psychological resilience for success. The combination of physical attributes such as agility, speed, and endurance (Bishop, 2012; Buchheit and Laursen, 2013; Griffin et al., 2006; Hanjabam and Kailashiya, 2015; Hegyi et al., 2019; Nuzzo et al., 2008), alongside psychological characteristics including motivation, anxiety management, self-confidence, positive thinking skills, and mental resilience (Arısoy and Pepe, 2021; Abdullah et al., 2016; Kalinowski et al., 2020; Olmedilla et al., 2019; Pepe et al., 2025), underscores the complex, multidimensional nature of football performance. This study examined how state desperation, psychological flexibility, and mental well-being mediate the relationship between stress and moral disengagement among football players, offering valuable insights into the psychological and ethical mechanisms underlying athletes’ behavior.

The demographic characteristics of the football players in this study highlight the interaction between physical and psychological attributes. The predominance of younger athletes reflects the sport’s emphasis on physical qualities such as speed and stamina, which are essential for performance. Conversely, the participation of more experienced players contributes to the team’s strategic depth, leadership, and tactical awareness. These findings are consistent with prior research showing that younger athletes tend to exhibit higher levels of moral disengagement, whereas maturity and experience enhance ethical reasoning and moral conduct in sport (Gentile et al., 2022). The relationship between age and moral reasoning supports the notion that accumulated experience promotes moral awareness and ethical decision-making (Gentile et al., 2022).

Moreover, the finding that 38% of the players possessed 9–14 years of experience indicates a group with considerable exposure to high-level competition. Such experience is associated with improved game intelligence, decision-making, and psychological maturity, all of which may influence ethical behavior. However, the results also align with previous studies suggesting that the pressures of elite-level competition can heighten psychological strain and increase vulnerability to moral disengagement (Reardon et al., 2019). Considering that only 11% of players had represented their national teams, most participants were still progressing toward elite performance levels. This underscores the importance of early psychological support for athletes approaching elite competition, where heightened pressure may adversely affect both mental well-being and ethical decision-making.

The results revealed a significant negative relationship between state desperation and psychological flexibility, emphasizing that heightened desperation impairs athletes’ capacity to adapt effectively to challenges. This finding corroborates the work of Ronkainen et al. (2024), who demonstrated that emotional distress undermines psychological flexibility and limits effective coping with adversity. Similarly, the negative correlation between state desperation and mental well-being indicates that as desperation intensifies, mental health deteriorates consistent with evidence linking emotional stress to increased risks of anxiety and depression (Ronkainen et al., 2024).

Furthermore, the positive relationship between state desperation and moral disengagement underscores the susceptibility of stressed athletes to rationalize unethical behavior. This finding aligns with previous research on moral disengagement mechanisms, such as the displacement of responsibility and the minimization of consequences (Corrion et al., 2009). These cognitive mechanisms, often observed in competitive sports, enable athletes to justify transgressive acts under pressure, thereby weakening ethical decision-making. The present results suggest that enhancing psychological flexibility may serve as an effective intervention to counter such tendencies. By improving athletes’ capacity to manage internal distress and remain committed to core values, psychological flexibility can reduce the likelihood of moral disengagement and foster more ethical conduct.

Consistent with earlier studies, the current findings showed that psychological flexibility positively correlates with mental well-being, indicating that flexible athletes manage stress more effectively and maintain a healthier psychological state (Ronkainen et al., 2024). The negative relationship between psychological flexibility and moral disengagement further suggests that flexibility not only enhances mental health but also acts as a protective factor against unethical behavior in competitive contexts.

The statistical analyses revealed that state desperation, psychological flexibility, and mental well-being together accounted for 20.8% of the variance in moral disengagement. Our model explains 20.8% of the dependent variable, moral disengagement (*R*^2^ = 0.208; Table 6). A moderate amount of explanation is expected in social and behavioral research because human behavior is multifactorial; however, the significant portion left unexplained by the model suggests the existence of other important variables that could influence moral disengagement (e.g., team moral climate, coach/youth attitudes, individual personality traits, on-field observational behavior, immediate match results, or economic pressures). Future research should aim to increase the model’s explanatory power by incorporating such contextual and personal variables.

Regression analysis also identified a significant relationship between state desperation and moral disengagement, indicating that emotionally distressed athletes are more prone to justifying unethical conduct. Although this model accounted for only 5.6% of the variance in moral disengagement, the result highlights the potential of moral disengagement to exacerbate psychological distress, including anxiety, burnout, and broader mental health challenges (Stanger et al., 2013). Addressing moral disengagement in high-stress environments could therefore serve as an effective means of improving both ethical decision-making and mental health outcomes among athletes.

The variability in state desperation observed across participants indicates that moral disengagement does not uniformly affect all athletes. This variability underscores the need to consider individual differences in stress perception, coping mechanisms, and moral reasoning. Future research should investigate additional predictors of state desperation and mental well-being to develop a more comprehensive understanding of these complex relationships. Tailored interventions that address individual differences in psychological resilience and moral cognition may be particularly effective in mitigating moral disengagement and enhancing ethical behavior.

In summary, this study demonstrates that state desperation negatively affects psychological flexibility and mental well-being, which in turn contribute to higher levels of moral disengagement. These findings highlight the pivotal role of psychological flexibility and mental well-being in reducing moral disengagement, particularly under competitive pressure. Interventions designed to strengthen psychological flexibility and promote mental health may therefore serve as effective tools to foster ethical behavior and enhance psychological functioning in athletes.

The present findings can be interpreted within Bandura’s Social Cognitive Theory, which conceptualizes moral disengagement as a cognitive mechanism that allows individuals to rationalize unethical behavior by temporarily suspending self-regulatory moral standards (Bandura, 1991, 2002). From this perspective, athletes experiencing heightened desperation may exhibit greater moral disengagement because emotional distress amplifies cognitive distortions that justify unethical actions. The positive relationship between desperation and moral disengagement observed in this study supports this theoretical proposition. Conversely, the negative association between psychological flexibility and moral disengagement is consistent with the idea that adaptive cognitive and emotional functioning safeguard individuals against moral self-justification. As articulated by Hayes et al. (2012), psychological flexibility enables individuals to tolerate discomfort, maintain value-driven actions, and respond adaptively to stress thereby reducing reliance on moral disengagement as a coping strategy. Integrating these theoretical insights, the serial mediation pattern identified in this study suggests that psychological flexibility and mental well-being jointly buffer the impact of desperation on moral disengagement. These findings emphasize the potential of Acceptance and Commitment Therapy based interventions to enhance athletes’ self-regulatory capacities and promote ethical decision-making in competitive sport environments.

## 5 Conclusion

The findings of this research underscore the pivotal role of psychological flexibility in shaping both the mental health and ethical decision-making of football players. The significant negative association identified between state desperation and psychological flexibility suggests that elevated levels of desperation considerably impair athletes’ emotional regulation capacities. In contrast, the positive relationship between psychological flexibility and mental well-being substantiates the notion that higher levels of flexibility are closely linked to enhanced psychological health. Collectively, these outcomes position psychological flexibility as a critical protective mechanism that fosters mental well-being while simultaneously mitigating the likelihood of moral disengagement.

Moreover, the observed relationship between moral disengagement and state desperation indicates that athletes experiencing heightened desperation may exhibit a greater propensity to rationalize or justify unethical conduct. Although the model accounted for 20.8% of the total variance, this proportion nonetheless signifies a meaningful contribution, highlighting the substantial impact of psychological variables on ethical orientations within the sporting context. Overall, the present findings highlight the importance of implementing targeted interventions to enhance psychological flexibility and mental well-being among athletes. Such initiatives hold promise for reducing moral disengagement, promoting ethical decision-making, and cultivating greater psychological resilience and responsibility in competitive sports environments.

### 5.1 Limitations of the study

This study was conducted only with football players who met the following criteria:

Between the ages of 18 and 35,

To have played football actively for at least 3 years in professional football clubs,

Currently participating in professional football leagues across Turkey in the 2024–2025 season.

The research was conducted on a sample group, which has the limitation of not representing the entire population. Therefore, similar studies with different sample groups should be conducted. Since this study was conducted only on Turkish professional football players, the results may not be generalizable to other cultural or league contexts.

The personal opinions and experiences of the study participants and their tendency to present themselves more positively may affect the generalizability and objectivity of the findings. Therefore, a similar study should be conducted experimentally and longitudinally. Data were collected entirely via self-report measures (online surveys), which introduces potential limitations such as social desirability bias and common method variance. Participants’ personal opinions, self-perceptions, and their tendency to present themselves more positively may have influenced the objectivity of the responses. To address this, future studies are recommended to use multi-source data collection methods (e.g., observations, reports from coaches or teammates).

The study utilized a cross-sectional correlational design; therefore, no claims of causal relationships between variables can be made, and only relationships observed at the same time point are reported. Furthermore, since the study was designed using a correlational survey design, the data were collected over a specific period, which limits the ability to establish causal relationships based on the data obtained.

The study utilized a cross-sectional design and relied entirely on self-report data. Mediation analysis was conducted purely from a statistical perspective, considering possible biases like common method variance and social desirability. The mediation analysis in this study was conducted from a statistical standpoint and should be interpreted considering these methodological limitations. The model explained 20.8% of the variance in the dependent variable (R^2^ = 0.208), indicating a moderate level of explanatory power. This also suggests that a large portion of the moral disengagement phenomenon (approximately 79%) could be influenced by other individual and contextual factors not measured in the present study such as team culture, coaching behaviors, and personality traits.

In addition, as the data were collected through self-reported questionnaires, there is a potential limitation related to the tendency of participants to respond in a socially desirable manner. In light of these limitations, future studies should utilize longitudinal designs, multi-source data collection, and replication with different national and cultural samples to validate and extend these findings.

### 5.2 Recommendations

Recommendations for this study.

Through this model,

The Turkish Football Federation can organize the necessary training to prevent moral disengagement and ensure fair play among football players.

Professional football clubs can offer programs to enhance psychological flexibility and mental well-being.

The Mindfulness Acceptance Commitment (MAC) approach developed by Gardner and Moore (2007) provides a practical framework for implementing such interventions in sport settings. Empirical studies have shown that MAC-based programs improve athletes’ attentional control, emotion regulation, and overall performance while reducing maladaptive cognitive responses under pressure (Gardner and Moore, 2007).

Coaches can improve performance by monitoring physical abilities and the players’ mental well-being.

Additionally, football players can receive support from psychological performance experts to develop their psychological profiles.

In addition, sport psychologists can design structured intervention programs grounded in Acceptance and Commitment Therapy (ACT) principles to enhance players’ psychological flexibility and reduce moral disengagement. Such programs may include mindfulness-based awareness training, values clarification exercises, and acceptance strategies that help athletes tolerate stress and align behavior with ethical values (Hayes et al., 2012).

Cognitive-behavioral workshops aimed at identifying and restructuring self-justifying thoughts that foster moral disengagement can also be implemented, following Bandura (1991, 2002) social cognitive framework. These workshops can help athletes recognize moral rationalizations, take responsibility for their actions, and strengthen moral self-regulation.

Furthermore, integrating psychoeducation sessions into team training schedules focused on ethical decision-making, emotional regulation, and coping with competitive pressure could support the prevention of moral disengagement and foster a culture of fair play.

Moreover, implementing ethics-focused awareness programs by the Turkish Football Federation can help players identify and challenge self-justifying cognitive distortions, thereby strengthening their moral accountability and fair play attitudes (Shields and Bredemeier, 2007).

Professional football clubs are also encouraged to integrate resilience and coping-skills training modules addressing emotional regulation, stress tolerance, and mental recovery to foster athletes’ ability to sustain performance under pressure (Fletcher and Sarkar, 2016).

In addition, coaches and sport psychologists can organize empathy- and communication-based workshops to cultivate team cohesion, interpersonal understanding, and prosocial behaviors that counteract moral disengagement (Kavussanu, 2008).

Finally, the incorporation of mindfulness-based mobile applications and digital ACT interventions can provide continuous and flexible access to mental training resources, promoting long-term development of psychological flexibility beyond formal sessions (van Emmerik et al., 2018).

### 5.3 Recommendations for future studies

The individual views of participants and their tendency to present themselves in a more favorable light may affect the generalizability of the findings. Therefore, experimental and longitudinal studies like the current one should be conducted.

Examining the effects of psychological flexibility and moral disengagement on mental well-being in athletes from a broader and more diverse range of sports could further enhance the generalizability of the results.

Additionally, conducting a study like this in different cultural contexts would help determine the impact of cultural factors. Future research can explore additional relationships mediated by mental well-being, thereby advancing our understanding of athletes’ psychological performance by addressing other psychological variables that may affect mental well-being.

Future research should also test the efficacy of targeted intervention programs designed to enhance psychological flexibility and moral reasoning among athletes. Randomized controlled trials incorporating ACT-based training or moral reasoning interventions could provide stronger causal evidence for reducing moral disengagement and improving ethical performance in sport.

## Data Availability

The data supporting the conclusions of this article will be made available by the authors, upon request. Requests to access these datasets should be directed to Mustafa Can Koc, cankoc_01@hotmail.com.
