# Supplementary material for: The psychological foundations of moral disengagement: the dynamic relationships between state desperation, psychological flexibility, and mental well-being
Source: Front Psychol. 2026 Jan 5;16:1716411. doi: 10.3389/fpsyg.2025.1716411 (PMC12813158; doi:10.3389/fpsyg.2025.1716411)
Supplement: Supplementary file 1 [file Supplementary_file_1.docx]

**Appendix A**

Exploratory factor analyses of the scales are given below.

**Table 1**

*The State Desperation Scale (SDS) factor analysis results*

| Items | Emotion | Motivation |
| --- | --- | --- |
| Item 1 | .721 |  |
| Item 2 | .821 |  |
| Item 3 | .602 |  |
| Item 4 | .801 |  |
| Item 5 | .726 |  |
| Item 6 |  | .719 |
| Item 7 |  | .605 |
| Item 8 |  | .708 |
| Item 9 |  | .418 |

**Table 2**

*Psychological Flexibility Scale (PFS) factor analysis results*

| Items | Value and Behavior in Line with Value | Being in the Moment | Acceptance | Contextual Self | Dissociation |
| --- | --- | --- | --- | --- | --- |
| Item 1 | .463 |  |  |  |  |
| Item 2 | .535 |  |  |  |  |
| Item 3 | .596 |  |  |  |  |
| Item 4 | .503 |  |  |  |  |
| Item 5 | .728 |  |  |  |  |
| Item 6 | .754 |  |  |  |  |
| Item 7 | .588 |  |  |  |  |
| Item 8 | .672 |  |  |  |  |
| Item 9 | .539 |  |  |  |  |
| Item 10 | .744 |  |  |  |  |
| Item 11 |  | .514 |  |  |  |
| Item 12 |  | .408 |  |  |  |
| Item 13 |  | .488 |  |  |  |
| Item 14 |  | .535 |  |  |  |
| Item 15 |  | .881 |  |  |  |
| Item 16 |  | .518 |  |  |  |
| Item 17 |  | .719 |  |  |  |
| Item 18 |  |  | .833 |  |  |
| Item 19 |  |  | .841 |  |  |
| Item 20 |  |  | .832 |  |  |
| Item 21 |  |  | .640 |  |  |
| Item 22 |  |  | .644 |  |  |
| Item 23 |  |  |  | .648 |  |
| Item 24 |  |  |  | .698 |  |
| Item 25 |  |  |  | .451 |  |
| Item 26 |  |  |  |  | .642 |
| Item 27 |  |  |  |  | .808 |
| Item 28 |  |  |  |  | .579 |

**Table 3**

*The Warwick-Edinburgh Mental Well-Being Scale (WEMWBS) factor analysis results*

| **Items** | **The Warwick-Edinburgh Mental Well-Being** |
| --- | --- |
| Item 1 | .670 |
| Item 2 | .537 |
| Item 3 | .756 |
| Item 4 | .848 |
| Item 5 | .540 |
| Item 6 | .942 |
| Item 7 | .475 |
| Item 8 | .437 |
| Item 9 | .430 |
| Item 10 | .759 |
| Item 11 | .538 |
| Item 12 | .591 |
| Item 13 | .471 |
| Item 14 | .621 |

## Table 4

## *Moral Disengagement in Sport Scale-Short (MDSS-S) factor analysis results*

| **Items** | **Moral Disengagement in Sport Scale-Short (MDSS-S)** |
| --- | --- |
| Item 1 | .570 |
| Item 2 | .985 |
| Item 3 | .827 |
| Item 4 | .450 |
| Item 5 | .998 |
| Item 6 | .995 |
| Item 7 | .990 |
| Item 8 | .996 |
